# Thiazides as an additional antiproteinuric treatment in young patients with Alport syndrome

**DOI:** 10.1093/ckj/sfaf008

**Published:** 2025-01-13

**Authors:** Valentine Gillion, Nathalie Godefroid, Kathleen Claes, Nada Kanaan

**Affiliations:** Department of Nephrology, Cliniques universitaires Saint-Luc, Brussels, Belgium; Institut de Recherche Expérimentale et Clinique, Université catholique de Louvain, Brussels, Belgium; Institut de Recherche Expérimentale et Clinique, Université catholique de Louvain, Brussels, Belgium; Department of Pediatric Nephrology, Cliniques universitaires Saint-Luc, Brussels, Belgium; Department of Nephrology, University Hospital Leuven, Leuven, Belgium; Department of Nephrology, Cliniques universitaires Saint-Luc, Brussels, Belgium; Institut de Recherche Expérimentale et Clinique, Université catholique de Louvain, Brussels, Belgium

To the Editor,

Thiazides have been used for years as antihypertensive drugs with a beneficial effect on cardiovascular morbidity and mortality [1]. Additionally, thiazide diuretics may have an antiproteinuric effect. Indeed, the CLICK Trial not only recently challenged the belief that thiazides are ineffective for treating hypertension in advanced chronic kidney disease, but also demonstrated a 52% reduction from baseline in the urinary albumin to creatinine ration in the chlorthalidone group at week 12, compared to a 4% reduction in the placebo group [2].

Here we report the effect of adding hydrochlorothiazide 12.5 mg to stable doses of angiotensin-converting enzyme inhibitors or angiotensin II receptor blockers on proteinuria in patients with X-linked Alport syndrome. Ten patients from 12 to 33 years old (eight men and two women) were retrospectively studied with a median baseline urinary protein/creatinine ratio (UPCR) 1.3 g/g and a median baseline estimated glomerular filtration rate (eGFR) of 90.5 ml/min/1.73 m^2^. (Supplementary Table 1) At month 6, the mean UPCR reduction was 34.2% (Fig. 1) and eGFR was stable. No electrolyte disturbance was reported (hyponatremia or hypokalemia) and tolerance was excellent. This old, affordable, and well-known medication is widely available and should be considered to minimize proteinuria and consequently slow the progression of kidney disease in Alport syndrome. This combination therapy will probably also be effective when used with other medications such as inhibitors of SGLT2 and/or finerenone. This warrants future investigations.

**Figure 1:**
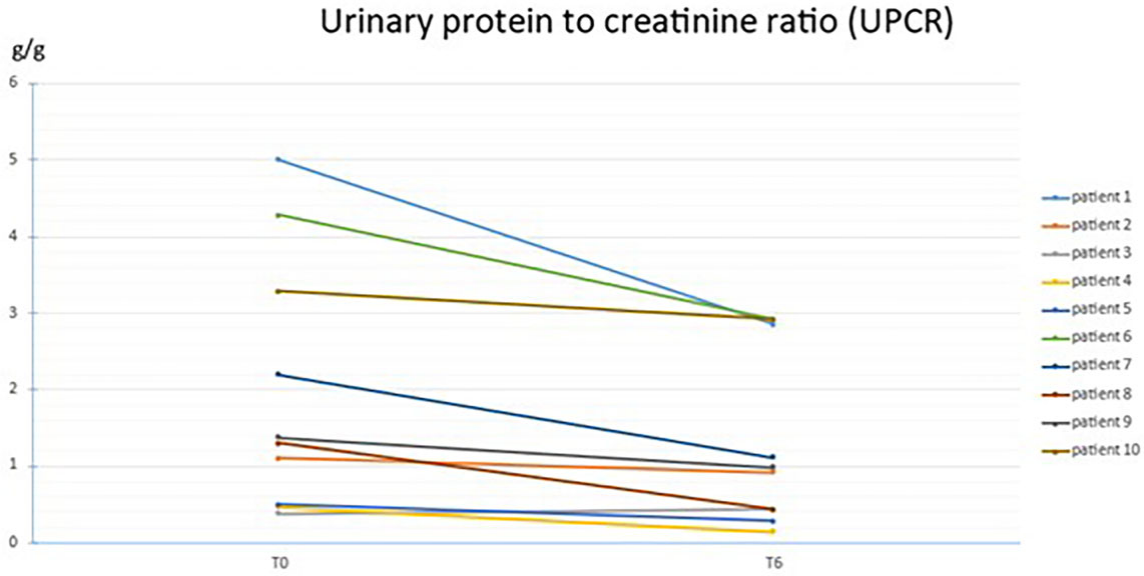
Evolution of UPCR at month 6 with the addition of 12.5 mg of hydrochlorothiazide.

## Supplementary Material

sfaf008_Supplemental_File
