# Supplementary figures and images for: Thiazides as an additional antiproteinuric treatment in young patients with Alport syndrome
Source: Clin Kidney J. 2025 Jan 13;18(3):sfaf008. doi: 10.1093/ckj/sfaf008 (PMC11997429; doi:10.1093/ckj/sfaf008)

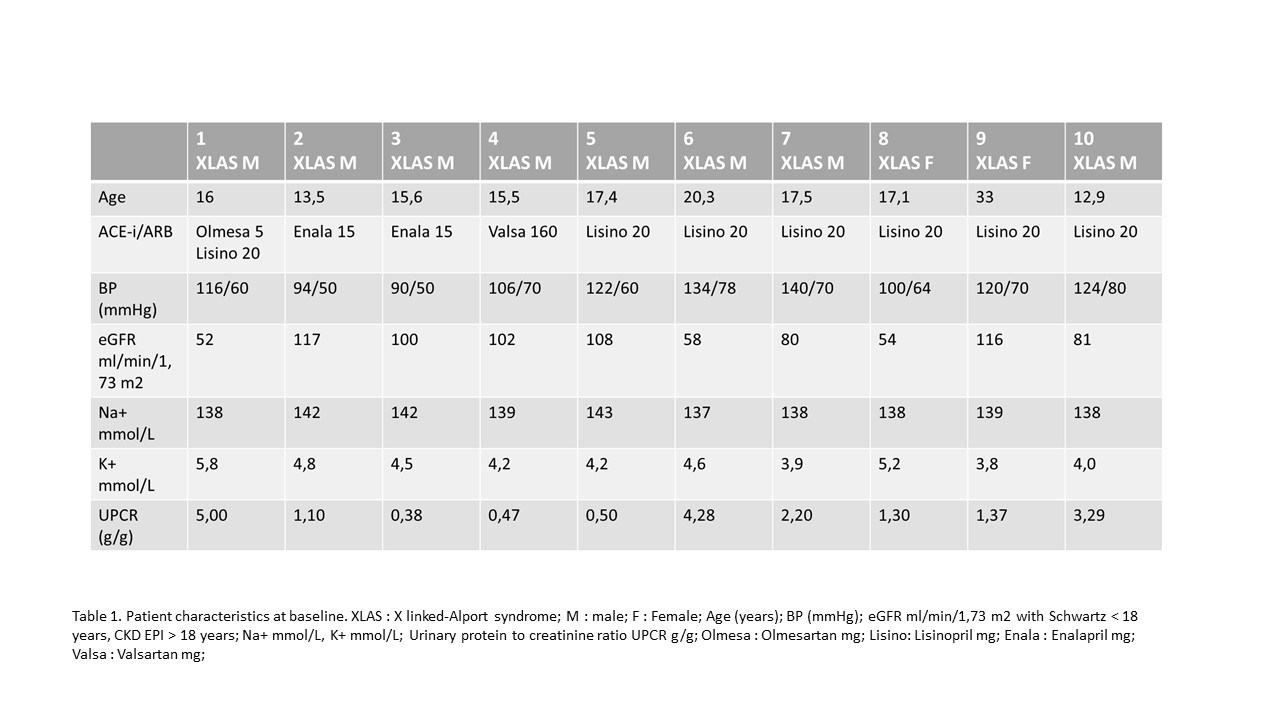

Supplement: sfaf008_Supplemental_File [file sfaf008_supplemental_file.jpeg]
